# Sharing Clinical Notes and Electronic Health Records With People Affected by Mental Health Conditions: Scoping Review

**DOI:** 10.2196/34170

**Published:** 2021-12-14

**Authors:** Julian Schwarz, Annika Bärkås, Charlotte Blease, Lorna Collins, Maria Hägglund, Sarah Markham, Stefan Hochwarter

**Affiliations:** 1 Department of Psychiatry and Psychotherapy Immanuel Klinik Rüdersdorf Brandenburg Medical School Theodor Fontane Rüdersdorf Germany; 2 Center for Health Services Research Brandenburg Medical School Theodor Fontane Rüdersdorf Germany; 3 Department of Women’s and Children’s Health Uppsala University Uppsala Sweden; 4 General Medicine and Primary Care Beth Israel Deaconess Medical Center Boston, MA United States; 5 Social Science Research Unit University College London London United Kingdom; 6 Department of Biostatistics & Health Informatics Institute of Psychiatry, Psychology & Neuroscience King’s College London London United Kingdom; 7 Department of Computer Science Norwegian University of Science and Technology Trondheim Norway

**Keywords:** electronic health record, open notes, user involvement, patient advocacy, patient portal, patient rights, collaborative health care, participation, coproduction, system transformation, health care reform

## Abstract

**Background:**

Electronic health records (EHRs) are increasingly implemented internationally, whereas digital sharing of EHRs with service users (SUs) is a relatively new practice. Studies of patient-accessible EHRs (PAEHRs)—often referred to as *open notes*—have revealed promising results within general medicine settings. However, studies carried out in mental health care (MHC) settings highlight several ethical and practical challenges that require further exploration.

**Objective:**

This scoping review aims to map available evidence on PAEHRs in MHC. We seek to relate findings with research from other health contexts, to compare different stakeholders’ perspectives, expectations, actual experiences with PAEHRs, and identify potential research gaps.

**Methods:**

A systematic scoping review was performed using 6 electronic databases. Studies that focused on the digital sharing of clinical notes or EHRs with people affected by mental health conditions up to September 2021 were included. The Mixed Methods Appraisal Tool was used to assess the quality of the studies. The PRISMA (Preferred Reporting Items for Systematic Reviews and Meta-Analyses) Extension for Scoping Reviews guided narrative synthesis and reporting of findings.

**Results:**

Of the 1034 papers screened, 31 were included in this review. The studies used mostly qualitative methods or surveys and were predominantly published after 2018 in the United States. PAEHRs were examined in outpatient (n=29) and inpatient settings (n=11), and a third of all research was conducted in Veterans Affairs Mental Health. Narrative synthesis allowed the integration of findings according to the different stakeholders. First, SUs reported mainly positive experiences with PAEHRs, such as increased trust in their clinician, health literacy, and empowerment. Negative experiences were related to inaccurate notes, disrespectful language use, or uncovering of undiscussed diagnoses. Second, for health care professionals, concerns outweigh the benefits of sharing EHRs, including an increased clinical burden owing to more documentation efforts and possible harm triggered by reading the notes. Third, care partners gained a better understanding of their family members’ mental problems and were able to better support them when they had access to their EHR. Finally, policy stakeholders and experts addressed ethical challenges and recommended the development of guidelines and trainings to better prepare both clinicians and SUs on how to write and read notes.

**Conclusions:**

PAEHRs in MHC may strengthen user involvement, patients’ autonomy, and shift medical treatment to a coproduced process. Acceptance issues among health care professionals align with the findings from general health settings. However, the corpus of evidence on digital sharing of EHRs with people affected by mental health conditions is limited. Above all, further research is needed to examine the clinical effectiveness, efficiency, and implementation of this sociotechnical intervention.

## Introduction

### Background

Electronic health records (EHRs) are implemented throughout health care as important tools for documenting and coordinating care within and across health care provider organizations. In the past 2 decades, secure patient portals have provided service users (SUs) opportunities to access certain information from the EHR and interact with health care providers. Administrative functions, such as appointment booking and prescription renewals, are common, but giving SUs access to the full clinical content of the EHR has been more controversial.

Although patient-accessible EHRs (PAEHRs) contain many different types of information [1], one of the more disputed functions has been providing SUs access to clinical notes or narrative visit reports. This practice is often referred to as *open notes* [2,3]. Open notes can be considered an essential part of any PAEHR. In some countries, for example, in Sweden [4], Norway [5], Finland [6], and Estonia [7], nationwide PAEHR services, including open notes, are offered to most adult citizens.

The legislation for giving SUs access to their EHRs is in place in many countries. In the European Union, most of the member states provide patients access to their EHRs; however, the level of access varies between member states. Legally, the SUs’ access to the EHR is usually covered by general data protection rules [8]. In the United States, since April 2021, a new federal rule mandates that all patients be offered access to their EHRs, including the narrative notes written by clinicians [9]. Notably, this rule encompasses the sharing of notes in psychiatry but excludes psychotherapy notes [10].

However, the sharing of mental health notes remains controversial. Health care professionals (HCPs) may be unclear when it is appropriate to *close* access or to hide aspects of documentation from patients. For example, in the United States, *information blocking* is permitted if doing so “will substantially reduce the risk of harm” to an SU or another person [11]. It is at the discretion of licensed HCPs to determine what constitutes a substantial risk *in the context of a current or prior clinician-patient relationship*. In such cases, the rules specify that risks must reach a clinical threshold well beyond the patient being upset [10]. However, as noted, these rules leave considerable latitude for interpretation, and so far, there are no clear procedures for monitoring or auditing clinicians’ decisions [12].

Moreover, beyond legal rulings, many HCPs report concern that SUs will become anxious or confused if they are offered access to their PAEHR [3,13]. HCPs also report concerns that providing access to SUs will reduce clinicians’ autonomy [14-17] and encroach on the quality of documentation [5,13-20]. Fears about additional workplace burdens [13,14,16-18,21,22], increased time spent responding to SUs’ anxieties, or misunderstandings about their clinical documentation [13,14,16,17,23] are additional concerns.

To date, between 2012 and 2021, 7 reviews have explored the effects of PAEHRs [24-30]. In 2020, a systematic review and meta-analysis found that adult patients’ access to EHRs was effective in reducing hemoglobin A_1c_ levels and could improve patient safety. However, the authors concluded that more methodologically robust studies were necessary to increase meta-analytic power and to evaluate the effects of access in different health care domains [30]. Similarly, in 2021, a Cochrane database systematic review found that the effects of patient access compared with usual care were uncertain [25].

In mental health, sharing SUs’ health records and the use of PAEHRs are also topics for further development and research. Although the implementation of PAEHR in mental health is similar to other health care domains, the perspectives, expectations, and experience in mental health can be of different natures. For example, the fear of unexpected consequences led to a *shadow record* in Norway [5]. Several studies have further identified that within mental health, there are strong divergent views, expectations, and concerns based on either the SUs’ conditions or the HCPs’ professional role, with psychiatrists holding the most negative attitude toward open mental health notes [5,13,17,20]. We also observed that many studies focused on whether the use of PAEHR can be harmful to SUs; this theme will be discussed in the *Special Challenges in MHC* section in more detail.

### Context and Scope for the Review

This is the first review to evaluate studies of PAEHR specifically among SUs affected by mental health conditions. In light of the new ruling in the United States and advances in patient access in the Nordic countries, evaluating the effects of PAEHR in mental health care (MHC) is particularly timely. As previous publications have stressed, PAEHR in MHC raises new practice dilemmas [10,31] but might also offer new opportunities to empower patients [32]. In the era of transparency, HCPs must now balance respect for patient autonomy and open and transparent information disclosures with duties to prevent patient harm. Persons with mental health conditions represent a vulnerable patient population, and there may be the potential to exacerbate perceptions of stigmatization or undermine the therapeutic alliance.

### Objectives

Considering the urgent need for greater clarity regarding best practices in this domain, our goal was to initiate a scoping review to explore what is understood about the effects of PAEHR among SUs, care partners, and HCPs. The study objective was to map the available findings on sharing EHRs with SUs affected by mental health conditions. Hence, we map the key concepts underlying the research area, (ie, mental health and neighboring fields) with the available evidence. The following research question is examined in detail: What is known from the existing literature about sharing EHRs or clinical notes with people affected by a mental health condition?

## Methods

### Scoping Review

Compared with the systematic review method, which is guided by a strongly focused research question, a scoping review aims to open up the spectrum of the available evidence on a relatively new field of research, so that its breadth and depth become visible [33]. A systematic scoping review was considered highly appropriate because of the lack of systematic reviews on the research topic and the exploratory nature of our research question.

The methodological framework for scoping reviews proposed by Arksey and O’Malley [33], its further development by Levac et al [34], and the Johanna Briggs Institute guidance on conducting systematic scoping reviews were applied in this work [35]. Accordingly, the following steps were performed: (1) identifying the research question; (2) identifying relevant studies; (3) study selection; (4) charting the data; and (5) collating, summarizing, and reporting the results, and consultation with stakeholders on how to report and integrate the study findings. As specified by Arksey and O’Malley, at least 2 reviewers (JS, AB, and SH) were involved in the study selection and analysis. To ensure reproducibility and traceability, a scoping review was carried out and prepared according to the PRISMA (Preferred Reporting Items for Systematic Reviews and Meta-Analyses) Extension for Scoping Reviews checklist to report our results (Multimedia Appendix 1) [33,34,36].

### Information Sources and Search Strategy

A literature search was conducted on April 16, 2021, and updated on October 2, 2021. A title and abstract search were carried out in 6 electronic literature databases: MEDLINE, Embase, Scopus, PsycINFO, Web of Science, and Google Scholar. The research question was based on three key concepts: (1) EHR, (2) sharing EHR with SUs, and (3) Mental Health, which were combined with the Boolean *AND* (Textbox 1). The search terms were adapted according to different databases. Subsequently, references of the found papers were scanned backward to find prior work that should be considered for the research topic [37]. This was followed by another forward search using Google Scholar to identify papers that cite the papers included in the review so far. Finally, in accordance with Haddaway et al [38], Google Scholar was used to track down *gray literature.*

**Key concepts of the search strategy.**

**Electronic health record search string**

- “open notes” OR “opennotes” OR “patient portal” OR “health record” OR “patient record” OR “psychiatric record” OR “clinical record” OR “health notes” OR “visit notes” OR “clinical notes” OR “psychotherapy notes”

**Sharing electronic health records with service users search string**

- Access OR show OR open OR share OR read OR engage OR participate OR participation

**Mental health search string**

- Mental OR psych*

### Eligibility Criteria

Inclusion and exclusion criteria (Textbox 2) were informed by the review process and were applied at the study selection stage. All studies were included in the review as long as the participants were involved in the process of sharing EHRs or were affected by mental health conditions. This included not only SUs and medical staff, but also stakeholders from administration, information technology, and health policy. However, only studies that focused on the digital sharing of health records with SUs were selected. Purely paper-based sharing of medical files was set as an exclusion criterion.

**Inclusion and exclusion criteria.**

**Inclusion criteria**

- Studies published up to September 30, 2021
- Studies in English
- No restrictions on the type of study
- Studies containing original empirical data
- Studies on service users affected by a mental health condition (>18 years)
- Studies on care partners or family members of people affected by a mental health condition
- Studies on health care providers
- Studies on policy stakeholders
- All health care settings
- No location restrictions

**Exclusion criteria**

- Gray data (Websites, tweets, and blogs)
- Paper-based sharing of patient files
- Pediatric and adolescent health care settings

Because people affected by mental health issues are treated at several other institutions in addition to psychiatric facilities (eg, outpatient psychotherapists and primary care by general practitioners), where sharing EHRs are also a common practice—at least in some countries—it was decided not to narrow down the search to individual medical specialties but to include all areas in which people affected by mental health conditions are treated.

All study types and designs were considered in this review. Search criteria were designed to include formally published peer-reviewed articles and selected grey literature (eg, dissertations and book chapters) as long as they contain original empirical data. *Gray data* such as websites, tweets, and blog posts were excluded. Studies conducted up to the end of September 2021 were eligible for inclusion.

### Selection of Studies

The search results were exported from the respective search engines, merged in a Microsoft Excel table (columns: author, year, title, and abstract), and duplicate entries were removed. Study titles and abstracts were screened independently by 2 reviewers (JS and SH) using predefined eligibility criteria. To select mental health populations that were treated outside of mental health settings, all studies dealing with PAEHRs were initially selected for title screening. If mental health populations were named in the abstract, corresponding publications were included. Papers were excluded when the abstracts were not available. If it was not possible to decide on the suitability of a paper based on the abstract, the full text was assessed. The screening results were then discussed and consented to by the reviewers. As part of this iterative process, the full texts of the preselected studies were read. The decision to exclude individual studies was made at the level of the full text.

### Data Extraction and Management

The research team developed a standardized template to extract and chart relevant data from the included studies [5,12-23,39-56]. The following parameters were recorded in detail: reference ID, authors, year, country, design, sample, participants, treatment setting and medical specialty, study purpose, and a summary of the results. The data were extracted by JS and AB, and checked for accuracy and completeness by CB (see Multimedia Appendix 2 [5,12-23,39-56] for more information).

### Quality Assessment of Studies

The Mixed Methods Appraisal Tool (MMAT) was used to assess the quality of the included studies [57]. This tool was developed for quality assessment in systematic reviews that comprise qualitative, quantitative, and mixed method studies. It was successfully tested for reliability and efficiency in systematic reviews [58]. The evaluation of the MMAT is based on criteria that are specific to the method used and includes the following: the suitability and rigor of the methods used, control of confounding factors, minimization of selection bias, and consideration of limitations. The MMAT grading was carried out by 2 researchers (AB and JS) independently of one another and consented to their results. When no agreement could be reached regarding the assessment, a third researcher (SH) was consulted. One study was excluded from the analysis owing to a low-quality score (Results section). Because of the limited informative value, the mean value of the MMAT of all included studies was not calculated. Instead, a detailed description of the quality of the included studies is provided based on the MMAT grading results. A comprehensive presentation of the individual ratings of each criterion can be found in Multimedia Appendix 3 [5,12-23,39-56].

### Qualitative Analysis and Synthesis

The results of all included studies were compiled and analyzed. The results were then analyzed independently by 2 researchers (AB and JS) using thematic analysis (Braun and Clarke [59]). In this process, the analytic material was increasingly summarized, and key themes were identified to organize the study results. The results of this synthesis process were discussed and approved by the entire research team.

### SUs’ Involvement Statement

The review was neither coproduced nor carried out with the participation of SUs with lived experience of mental distress or any form of *Patient and Public Involvement*. However, after completion of the review, 2 researchers (LC and SM) with lived experience were invited to critically comment on the paper from an SU’s perspective. The commentaries are attached in the Multimedia Appendix 4.

## Results

### Study Selection

**Figure 1:**
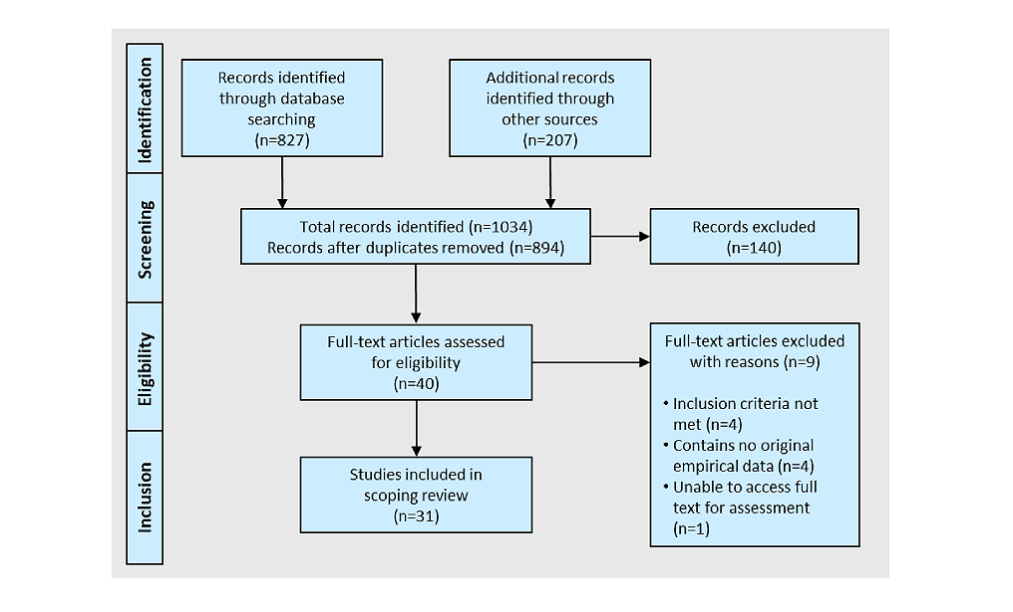
PRISMA (Preferred Reporting Items for Systematic Reviews and Meta-Analyses) flow diagram adapted from Moher et al [60].

In total, 1034 records were identified—827 (79.98%) records from database searching, 207 (20.01%) through other sources (198 from Google Scholar, 9 through communication with authors and consultation with experts). After removing duplicates, 86.46% (894/1034) entries remained for title, abstract, and keyword screening. This step reduced the selection by a total of 854 to 40 records that were then subjected to a full-text eligibility check. Finally, 2.99% (31/1034) of entries that met the inclusion criteria of this review article were retained. The study selection process is shown in the PRISMA flow diagram (Figure 1) adapted from Moher et al [60].

### Basic Characteristics of the Body of Evidence

These studies mainly used qualitative methods or surveys. A randomized controlled trial design was used once among the observational studies. Instead of well-established outcome measures, self-developed and unvalidated questionnaires were predominantly used in the surveys. A comprehensive overview of the basic parameters of the included studies is presented in Table 1.

**Table 1:** Basic characteristics of the included studies (N=31).^a^

Parameter			Total, n (%)		References
**Study design**					
	Qualitative	10 (32)		[12,15,16,18,21-23,39,50,51]	
	Survey	9 (29)		[5,13,14,17,40,43,45,48,49]	
	Mixed method	4 (13)		[20,46,47,55]	
	Descriptive	2 (6)		[53,54]	
	Cohort	2 (6)		[41,52]	
	Intervention	4 (13)		[19,42,44,56]	
	Randomized controlled trial	1 (3)		[44]	
**Publication year**					
	2012-2014	1 (3)		[44]	
	2015-2017	6 (19)		[13,15,19,39,47,56]	
	2018-2020	18 (58)		[5,14,16-18,20-22,40-43,45,46,49,52-54]	
	2021 and onwards	6 (19)		[12,23,48,50,51,55]	
**Country**					
	Australia	1 (3)		[21]	
	Canada	3 (10)		[20,45,56]	
	Netherlands	1 (3)		[23]	
	Norway	3 (1)		[5]	
	Sweden	5 (16)		[14,16,17,50,55]	
	United Kingdom	2 (6)		[47,51]	
	United States	8 (58)		[12,13,15,18,19,22,39-44,46,48,49,52-54]	
**Study participants**					
	Service users	17 (55)		[19,23,39-41,43-49,51-54,56]	
	Veterans	5 (16)		[39-41,43,52]	
	Health care professionals	16 (53)		[5,13-23,42,53-55]	
	Relatives	1 (3)		[45]	
	Policy stakeholders	2 (6)		[12,50]	
**Treatment setting**					
	Inpatient	11 (35)		[5,14,16,17,23,45,47,50,52,55,56]	
	Outpatient	29 (94)		[5,13-23,39-50,52-56]	
	Not applicable	1 (3)		[51]	
**Clinical field**					
	Psychotherapy	4 (13)		[18,46,53,54]	
	Psychiatry	13 (42)		[5,14,16,17,19,20,23,45,47,48,50,55,56]	
	Veterans Affairs Mental Health	9 (29)		[13,15,22,39-43,52]	
	Somatic (General practitioner, Primary Care and other)	6 (19)		[5,21,44,48,49,51]	
	Not applicable	1 (3)		[12]	

^a^Individual papers can be assigned to the various subparameters at the same time, which means that percentages of over 100% can be achieved.

### Search Results

The results of the qualitative analysis and synthesis are presented thematically based on the main categories formed. The identified categories were as follows: (1) SUs’ experiences of reading mental health notes (positive and negative), (2) experiences of care partners, (3) HCPs’ experiences of sharing mental health notes (benefits and risks), and (4) views of policy stakeholders and experts.

### SUs’ Experiences

#### Overview

SUs’ attitudes and experiences toward OpenNotes were evaluated in about half of the papers. This included whether reading OpenNotes had any effect on SUs’ MHC, such as if the patient-clinician relationships were affected. In most papers, the participants were diagnosed with one or more mental disorders, such as posttraumatic stress disorder (PTSD) [22,39-41,43,52], bipolar disorder, psychotic spectrum disorder [22,39-41,43,48,52-54], personality disorder [40,43,44,47], substance use disorder [40,43,44,47,52], and major depressive disorder [22,39,41,43,46,53]. Other mental health issues among the participants were depression (unclear to what severity or type) [40,44,52], mild or atypical depression [46], anxiety disorder [41,43,47,52,53], military sexual trauma [52], and other mental illnesses within the International Classification of Disease-10 codes F00-F99 [48]. In 5 papers, mental health diagnoses or issues were not highlighted [19,45,49,51,56]. The most frequent care settings were outpatient and inpatient psychiatry, outpatient veterans’ mental health, outpatient psychotherapy, and primary care settings.

#### Positive Experiences

Positive experiences were reported to the greatest extent in all studies [19,22,39,40,45-47]. In one study, 94% (108/115) of all study participants reported that being able to read therapy notes on the web is a good idea [46]. In another study, most participants reported that they were extremely to moderately positive about open mental health notes [40]. SUs’ reported that they wanted to continue having web-based mental health notes available [19,46,47]. It was reported that open notes within MHC increased feelings of validation [46], SUs’ sense of control of their care 48.9% (87/178) [40,46], 82% (43/52) [19], 93% (372/400) [49], and helped SUs to understand potential side effects of medications, as well as to remember to take their medications [19,48,49,52]. One survey analysis examined SUs’ experiences with open notes by comparing persons with serious mental health diagnoses (defined as major depressive, psychotic, schizoaffective, or bipolar-related disorders), persons with other mental health diagnoses, and those with no mental health diagnoses [48]. The study found that 20% of SUs with serious mental health diagnoses, and 18% with other mental health diagnoses reported that they were more likely to adhere to their medications after reading their notes, compared with 14% of persons with no mental health diagnosis. The study also reported that among SUs with serious mental health diagnoses, the majority reported a better understanding of why medications were prescribed (67%), feeling more comfortable (65%), and more in control (67%) of their medications, and that notes helped answer questions about their medications (60%) [48].

In one self-reported web-based survey study from the United States, more than half of the study participants (total n=52) reported that open notes helped them to remember the plan for their MHC [19]. Other studies report that being able to access and read notes is extremely important for SUs to better take care of themselves [46,49]. Studies also report that open notes increased SUs’ understanding of their mental health [19,49] and awareness of their diagnosis [45]. In addition, SUs reported being better prepared for their visits [46,49], and the odds of attending their scheduled appointments increased by 67% after portal implementation [56]. A study at the Department of Veterans Affairs (VA) reported that the most frequently used features were medication refill, appointment view, secure messaging with the HCP, and Blue Button (eg, allowing the SUs to share their VA care documentation with a non-VA health care provider) [52].

Several papers have reported that open notes within MHC help SUs gain trust in their clinicians [39,40,46] and improve transparency [46]. SUs emphasized the importance of talking openly and upfront to each other [22,39]. In one of the semistructured interview studies conducted in the United States, SUs emphasized the importance of obtaining an overall picture of their mental health via the notes and of detailed notes that thoroughly summarized each visit. Notes that were written in this way reportedly increased feelings of being understood by their clinician. Notes that embraced strengths and progress in treatments also reportedly improved health and increased feelings of being valued and supported by their clinician [22]. The accuracy of the notes was also identified as a reason why SUs wanted to read their notes [19,22,45,46]. Health users desired the opportunity to ensure that no errors occurred and that the description of the visit was correct [19,45,46]. In one cross-sectional survey study conducted in the United States, 94% of the SUs (total n=108) reported that the description of the visits in the notes usually or always conformed to their visit [46]. Another survey study conducted in the United States found that SUs diagnosed with PTSD were more likely to report having read their notes [43]. A Canadian study reported improved recovery among SUs that use a patient portal according to Mental Health Recovery Measures. The study also reported an 86% decrease in the number of requests for health information among SUs [56].

Two studies have evaluated the impact of EHR use on in-session behavioral treatment with a computer screen facing the SU [53,54]. SUs reported that EHR use during their appointment did not impact communication and increased collaboration during their planning [53], and that collaborative documentation endorsed a strong therapeutic relationship [54].

In a feasibility study conducted in the United Kingdom, participants with severe mental illness monitored their health. The participants self-monitored and interactively input their health information into the EHR, which allowed them to self-monitor and become interactive with the service. The participants reported that the interactive part was most useful because they could monitor their mood over time, allowing them to better understand their illness [47]. In one cross-sectional randomized controlled trial, the hypothesis of mental health or substance use conditions as a possible barrier to engagement with web-based health information was examined. The results found that a mental health diagnosis was not a barrier to the ability to use a PAEHR [44]. The same results were reported in a mixed method study with a survey and interviews, where SUs with a severe mental health diagnosis reported the benefits of using a PAEHR to self-monitor health outcomes, which contributed to experienced improvements in well-being over time [47]. Another study reported SUs’ positive experiences of a web-based educational program of open notes, which appeared to improve patient activation, trust in their clinician, and efficacy in health care interactions. Schizophrenia spectrum disorder, bipolar disorder, older age, and a higher number of mental health visits were common variables that were significantly associated with these improvements [41].

In one study, 43.7% (45/103) SUs reported interest in providing portal access to a care partner, and 33% (34/103) participants were concerned about privacy breaches and cybersecurity [45]. Other papers reported that SUs shared or discussed their notes with others; the most frequent sharing or discussion was with a care partner (15/108, 13.9% [46]; 9/52, 17% [19].

#### Negative Experiences

Negative experiences have been reported in fewer papers and consistently on a smaller scale. Some SUs reported feeling judged and labeled [22,46], and 1 participant (total n=28) in a semistructured interview study described how the tone of the notes made him feel like being perceived as a complainer [22]. Some SUs felt offended by their notes (2/108, 1.8% [46]; 5/52, 9% [19]), or offended and disrespected by the tone of their notes [39]. Studies also reported that some health users experienced stress and worry when reading their mental health notes (14/178, 7.9% [40]; 9/108, 8.3% [46]; 2/52, 4% [19]; 32/400, 8% [49]), which caused some individuals to question the nature of documentation and therapy itself [19]. Feeling upset when reading mental health notes was also reported sometimes 17.9% (32/178) or often or always 7.9% (14/178), with the most frequent response “the notes make my problems seem smaller than they are” [40]. One study reported that health users found simple language to be preferable to medical terminology [22].

Studies have reported that SUs expressed feeling upset and worried when seeing inaccuracies in their notes or details with a lack of congruence between what the note said and their recollection of a visit [19,39,46]. Others felt confused and blindsided when discovering diagnoses in their notes that had not been discussed with them [22,39] and worried that errors could affect their mental health treatment [19,39]. Such incongruences in the notes contributed to strained trust in clinicians, as they experienced low transparency or lack of respect [22,39]. Some SUs also expressed concerns about privacy and confidentiality (15/108, 13.9% [46]; 9/52, 18% [19]; 164/400, 41% [49]). In addition, health SUs reported concern about who could access their mental health notes, and that medical appointment notes should be between them and their care providers [19,46]. One qualitative focus group study investigated SUs’ expectations of having access to their mental health notes. The participants emphasized the need to maintain confidentiality and expressed concerns about the security of the data systems. They wanted to have the choice to decide what information should be shared (eg, with other HCPs within the organization) and raised concerns about inaccurate notes and the need for transparency from their clinician regarding the content of the notes [51]. One self-reported cross-sectional survey study conducted at the Veterans Affairs Medical Center in the United States reported that SUs with PTSD diagnoses were more likely to experience negative emotions after reading their notes (no further explanation of why in the paper) [40].

#### Experiences of Care Partners

Only one study included care partners, such as family members and friends, as study participants in a cross-sectional survey (total n=7) [45]. Participants stated that to be able to support their family members it would be helpful and convenient to have access to the SU’s health record. They expressed an interest in accessing health records, messaging providers, receiving educational materials, appointment times, and self-assessments. Of the 7 participants, 5 (71%) expressed an interest in scheduling appointments and renewing medications.

#### HCPs’ Experiences

HCPs’ attitudes and experiences toward open notes in MHC were explored in about half of all papers. Study settings included veterans mental health (inpatient [22]; ambulatory [13]), inpatient and outpatient (1 large mental health hospital [20], 1 university hospital [15], 1 adult psychiatric clinic at a university hospital [14,16,17,55]), 2 ambulatory care settings [18,19], 1 primary health care [21], 2 health centers [53,54], and 1 MHC organization [23]. Psychiatrists, nurses, psychologists, therapists, and medical secretaries were the most common study participants.

#### Experienced Benefits

Unlike perceived risks, the HCPs’ perceived benefits of sharing mental health notes with SUs were reported in most studies to a minor extent. Mental HCPs believed that open notes contributed to better documentation [13,18,22], increased patient-clinician collaboration [13,22], and improved SU participation in care [13,15,19,23]. They also believed that open notes strengthened the patient-provider relationship [15,18,23], increased transparency [15,20], and increased feelings of trust [14,15]. A survey study from Norway reported that 2.39% (107/4477) mental HCPs believed that SUs in MHC had increased understanding of their diagnosis, treatment, and follow-up when reading their EHR [5].

One semistructured interview study with therapists, conducted in the United States, reported that the most significant impact of open notes was that it encouraged HCPs to be more sincere during visits and that it was easier to address difficult topics because communication was strengthened [18]. Transparent communication was reported to be important in maintaining patient-provider relationships and helpful when documenting potentially surprising and sensitive information such as diagnoses [15,18,22]. One self-reported survey study that evaluated the effects of a training program for mental health staff on open notes reported communication improvements, such as more frequently advising and educating SUs to access and read their notes, as well as more frequently asking SUs about questions and concerns regarding their notes [42]. One study evaluated the impact of EHR use on in-session behavioral treatment with a screen facing the SU, where HCPs confirmed the accuracy and acceptability of documentation with the SU to a large extent [54].

Notes that highlighted SUs’ strengths and treatment progress were perceived as necessary by mental HCPs [22]. These notes could help SUs gain control of their health and treatment [14,16,23], and also demonstrate that the HCPs heard and understood the SUs’ from a patient perspective [22]. Some papers reported that HCPs believed that collaborative notes could facilitate patient-centered care [15] and, in turn, that the power between care providers and SUs can be more equally distributed [15,21]. One full-population survey conducted before implementation in Sweden reported that mental HCPs believed that open notes within MHC would contribute to equal terms for all SUs, patient satisfaction would improve, and MHC would be both more efficient and safer [17]. In a full-population survey conducted after implementation in Sweden, mental HCPs believed that open notes improved SUs’ recall about their care plan better, helped them to be better prepared for visits, increased their understanding of their mental health condition, and strengthened health care SUs’ trust in them as clinicians [14].

#### Experienced Risks

Most papers reported mental HCPs’ anticipation of and experiences with open mental health notes; negative experiences and risks were reported on a larger scale, unlike positive experiences and benefits. The negative impact on SUs was a commonly expressed risk. Both before and after the experience of the practice, many HCPs believed that SUs would worry more [13,14,17], disagree with the content of their notes [13,17] or their diagnosis [13], be confused or offended [15,17,19,20], or that the assessment might be stigmatizing [15,19]. A Swedish full-population survey study reported that 25.5% (178/699) of the respondents experienced notes being more confusing than helpful for SUs [14], and that open mental health notes could make the care process less effective [16]. Other papers report respondents’ worry regarding patient disengagement from care [13,19] and increased clinical burden [13,14,16-18,20,21,23]. A Swedish full-population baseline study (total n=871) found that mental HCPs worried that visits would take significantly longer (35%), that they would spend more time addressing SUs’ questions outside of visits (40%), that they would spend more time dictating, writing, or editing notes (41.5%), and that they would be less candid in documentation (40.5%) after SUs are provided access to EHRs [17]. A survey study from Norway reported that 28.9% (1298/4477) of HCPs in psychiatric care did not report all relevant information in the EHR, and they reported spending more time writing notes (29%) [5]. In one United States survey study conducted in the Department of Veterans Health, about half of the total respondents (n=263) wanted open notes within MHC to be discontinued [13].

Many mental HCPs perceived a negative impact on the therapeutic relationship [15,18,20,22], as open notes may disconnect SUs from the in-person experience [15], and therefore, not facilitate discussions and the development of good relations during the visits [15,20]. A United States study, with semistructured interviews conducted at the Veterans Health Administration reported that HCPs’ concerns about how shifting patient-clinician power distribution affected their approach of providing care, such as how some SUs almost dictated how to write their notes and what information they should exclude [15]. Other papers report concerns about SUs’ lack of understanding of medical terminology documented in the notes, which could lead to misunderstandings and misinterpretations [14,16-19,23]. HCPs reported being less candid, less detailed [13,14,16-20,22], changing the tone of their documentation, and reported being afraid SUs’ might find errors and request changes [13,17,23]. Sharing sensitive information with SUs has been raised in some papers as a concern [19-23]. HCPs recommended excluding detailed information of traumatic experiences for privacy and safety reasons [22] and wanted to assess what to include in the notes on a case-by-case basis [19,20,22]. Issues of anonymity, privacy, and patient safety have also been raised as concerns [13,14,16-18,20,23,55], as HCPs reported not being able to protect SUs and the disclosure of third-party names [14,16,17,23]. One study evaluated the impact of EHR use on in-session behavioral treatment with a screen facing the SU, where HCPs were more likely to perceive in-session computing as more harmful to communication and computer use more disruptive than SUs [53].

In a pilot survey study conducted in the United States, mental HCPs offered SUs access to their mental health notes for 20 months. The study reported that the severity of illness, duration of treatment, and psychiatric diagnoses were critical variables in their selection, and psychotic, personality, cognitive, bipolar, and substance use disorders were excluded [19]. Another survey study conducted in Sweden found that mental HCPs experience personality disorders, psychosis, and paranoia as the most challenging SU groups to access and read their notes [14]. Studies have reported that the HCPs with the most negative attitude toward open mental health notes are psychiatrists [5,13,17,20], those working in acute care settings [20], nurse practitioners [13], psychologists [17], and medical secretaries [17].

### Views of Policy Stakeholders and Experts

Two studies included the views of policy makers [50] and experts [12] on open notes in MHC. The first study explored Swedish national and local policy regulations regarding SUs’ access to their psychiatric notes and to what extent they were offered access. Regional policies and regulatory documents were analyzed, and key stakeholder email interviews were conducted. The study reported that out of Sweden’s 21 self-governing regions, 17 (80%) shared adult psychiatry notes with SUs, 15 (71%) regions shared pediatrics and adolescent psychiatry notes, and 8 from forensic psychiatric care. Of the 6 regions that did not share notes from forensic psychiatric care, 2 (33%) planned to implement open notes, whereas 4 (67%) had decided to exclude open notes from this psychiatric care setting. All 17 regions shared psychiatry notes with both outpatients and inpatients. Immediate access to open notes was most common, followed by a 14-day respite period to provide access [50].

The latter study [12] was a web-based purposive survey of 70 experts on open notes drawn from 6 countries, including informaticians, clinicians, chief medical information officers, SUs, and patient advocates. Participants emphasized the importance of educating mental HCPs in writing notes and offering SU guidance on the risks and benefits of access. Experts suggested that HCPs should become more knowledgeable about patient terminology or use everyday language, and highlighted the importance of accurate, objective, and truthful notes. Experts addressed the need for guidance on how to describe sensitive and challenging topics in the notes. Recommendations included the need to train the staff on dealing with practice dilemmas specific to sharing mental health notes and dealing with possible disagreements [12].

### Quality of Included Studies

We reported the grading according to the recommendations by the authors of MMAT [57]. Overall, we found that the quality of the included studies was very high or high, with some exceptions. We found the findings of these studies to be valuable and included these in our review after a discussion between the reviewers (AB, JS, and SH). In contrast, we excluded 1 paper with very low MMAT grading, as it was a design paper providing little insight into our research question. Of the 31 studies included, 16 (51%) used quantitative analyses, with majority reporting results from the surveys. The mean response rate was mostly low to moderate. Hence, there was a moderate bias in the nonresponse bias in these studies. A detailed description of the included studies and an overall MMAT grading can be found in Multimedia Appendix 3.

## Discussion

### Principal Findings

PAEHRs in MHC are a fairly new field, so the available evidence is limited. However, the increasing number of studies carried out in recent years has confirmed the increasing interest in applying PAEHRs in the mental health sector. It is not surprising that most of the studies were conducted in the United States and Scandinavia, given the legal initiatives there and the implementation of OpenNotes that go beyond pilot projects. The results of the included studies show a clear predominance of positive experiences among SUs, who in turn face an excessive amount of perceived burden and fears on the part of the HCPs—an aspect that requires a closer look. The results also point to several practical and ethical challenges that reveal both structural barriers and resistance on the part of the HCPs to change their usual routines and abandon the previous routines in the transparent handling of medical documentation. The extent to which these findings are specific to the MHC field can only be clarified in comparison with research from general health care settings. Therefore, we will (1) compare findings across different medical fields, (2) compare stakeholders’ expectations and experiences with PAEHRs, (3) discuss the special challenges of PAEHRs in MHC settings, and finally, (4) deduce tasks for future research on PAEHRs in MHCs.

### Comparison With Non-MHC Settings

First, the corpus of evidence on PAEHRs in general health settings is significantly more extensive, which is reflected, for example, in the number of available reviews [25,27,61-63]. Although studies conducted in the mental health sector are primarily of an exploratory, qualitative, and descriptive nature, recent work from general health settings includes overall effectiveness studies often with randomized controlled designs [64]. Looking at the examined outcomes, improvements in medication adherence, disease awareness, self-management of the illness, and a decrease in office visits were demonstrated for nonmental health users [65,66]. However, there are also several qualitative findings on the experiences of veterans with PAEHRs in general health settings that are very similar to the included evidence. For example, Woods et al [67] found that the use of PAEHRs improves patient-clinician communication and appointment recall and that SUs’ health literacy, understanding, and control of health issues were strengthened. Furthermore, veterans reported almost the same issues with PAEHRs as users affected by a mental health condition (eg, concerns about medicalized language in clinical notes and inconsistencies and errors in the documentation). This study is particularly comparable across medical fields, as the same access system *myhealthevet* was used.

### Negative Expectations Versus Positive Experiences

As stated above, the present findings show an imbalance between negative and positive views of HCPs (and SUs) with PAEHRs. For instance, the assumption that SUs would often disagree, feel offended, or stigmatized with notes written by their therapist predominates the staff perspective in the existing evidence. These results contrast with the predominantly positive experiences of the users, who often describe the notes as very precise and a reflection of the visit that took place [19,22,45,46]. A closer look reveals that the focus of several of the included studies was on expectations *before* having used the EHR, and a significantly lower proportion of HCPs were asked about their *actual experiences* with the EHR. At this point, a comparison with general health settings is useful, where PAEHRs were piloted and researched much earlier than in mental health settings: a qualitative study of HCPs’ and SUs’ expectations toward PAEHR carried out in 2005 showed a similar imbalance [68]. With increasing implementation and use experiences, HCPs’ views on PAEHRs seem to have become increasingly positive [69]. Beyond that, the introduction of innovations in health care that disrupts or changes HCPs’ routines often appears to be accompanied by skepticism and discomfort [70]. This aspect seems to be more intensified when it comes to innovations that aim to expand the power or influence of SUs in the treatment process. For instance, the introduction of second opinion programs in the early 2000s—to check a physician’s recommendation of a particular surgical intervention—led to considerable skepticism and reservations among physicians, whereas this quality assurance measure has been proven to be a helpful and accepted standard in various health systems [71,72]. A sensitive way of dealing with these resistances could be shown in one of the included studies, which evaluated a web-based educational program on OpenNotes [42]. The provision of training HCPs on how to share the EHR seems to be of fundamental importance to address fears and reservations and contrast them by the overall positive evaluation results.

### Special Challenges in MHC

Several of the included studies dealt with the question of whether PAEHRs could be harmful or disadvantageous for SUs with certain mental health issues or acute illness states and should therefore be limited. The findings of this study are ambiguous. Initially, none of the included studies revealed critical events, such as self-harm, suicide attempts, or other violent behavior in connection with PAEHRs. Although isolated negative effects from reading EHRs have been described, they do not seem to be related to specific mental health conditions. Some unspecified adverse effects have been described for SUs with PTSD in Veterans Affairs Mental Health and can therefore not be easily transferred to other MHC settings [40]. The concerns expressed by clinicians toward individuals with whom it has been hard to develop trusting relationships with, or are delusional or paranoid, or who are prone to violence could not yet be confirmed by any of the included observational studies, which included SUs as participants. These concerns seem to be comprehensible from a professional point of view; however, instead of denying access to a subset of patients or not informing them about the possibility of accessing their EHR, one of the included studies recommended offering an educational program on the use of PAEHR to learn about the benefits and misuses of reading therapy notes, and to discuss possible adverse side effects with each individual SU. Discussion about restricting access to the EHR to only a subset of SUs, however, can be considered contrary to the basic idea of coproduction and may lead to epistemic injustice [73]. This might also apply to partial access restrictions, such as sharing notes only on a case-by-case basis or the release of clinical notes after an acute mental crisis has subsided [31]. PAEHR is of overriding relevance in the field of MHC, where therapeutic decisions are often guided by clinicians’ individual experiences, which are applied to the individual case instead of a small-step procedure described in guidelines, unlike other medical disciplines, such as surgery or internal medicine. Therefore, it is important to make medical decisions as comprehensible and transparent as possible. Hence, it cannot be concluded from currently available evidence that restricting PAEHRs makes sense for certain groups of SUs. Conversely, users should be able to dispose of omissions or restrictions on the release of their EHR themselves, especially when it comes to particularly sensitive information that should not be accessible to family members, for example, in the event of intimate partner violence or sexual abuse. In this regard, further research should assess the needs of SUs to be able to develop evidence-based best practices [74].

### Limitations of Studies

Several important limitations arise from the studies reported in this scoping review. Most of the studies were based on surveys, and it is not known whether response biases affect findings. One-third of the included studies (8/31, 25%) were carried out in Veterans Affairs Mental Health settings, and half of these studies (4/8, 50%) selected American veterans as participants. As already discussed, this group is not necessarily comparable with the general population with regard to the distribution of psychiatric disorders and use behavior, which limits generalization.

Other studies examined PAEHR implementations in which only selected SUs were granted access to their EHR. In the Peck et al [19] study for example, treating clinicians decided for themselves which users were included or excluded from the intervention (PAEHRs). These results can also only be transferred to the population of psychiatric users to a very limited extent.

Much of the included evidence relates to OpenNotes, which is a self-described advocacy research unit that supports the dissemination of PAEHRs (note: not to be confused with VA OpenNotes, which is not connected or formally affiliated with the OpenNotes advocacy group). This implies a possible conflict of interest and increases the risk of positive bias in the results. Similarly, some authors contributed up to 10 of the 25 included studies, which increased the risk of not being able to replicate the findings [75]. However, because all included studies were subjected to a comprehensive methodological quality check in this review, the risk of these biases can be ranked as low.

### Future Research

In addition to the research gaps already mentioned, the present corpus of evidence is incomplete and needs to be extended. First, the predominantly exploratory findings must be quantitatively validated. Currently, there is a lack of studies examining the effects of PAEHRs on the basis of well-established psychological outcomes such as symptom severity, social functioning, and empowerment using controlled designs and including more participants over a longer period of follow-up, as this may increase the likelihood of detecting the effects of the intervention.

Second, there is no evidence on the efficiency of PAEHRs in MHC. Therefore, the exact treatment costs of SUs having access to their EHRs should be measured and compared to demonstrate the efficiency of this intervention. Approximately 60% of psychiatrists’ working time in acute settings is not patient-related [76]; thus, it should be investigated to what extent PAEHRs may increase the time spent on documentation or whether PAEHRs can reduce treatment costs in the long term by accelerating therapeutic processes.

Third, several EHR solutions contain interactive tools to promote self-management and monitor the mood or activities of the SUs’ everyday lives. Further research should explore the role and scope of these extensions in PAEHR. To understand psychiatric treatment as a fully coproductive process, the EHR should not only be accessible but also easy to use among SUs [77]. Methods of participatory design can help study this.

Fourth, most of the existing interventional studies have excluded persons affected by severe mental illness or those being (involuntarily) treated in inpatient settings, often for safety reasons. However, there is initial evidence for a meaningful application of PAEHRs in acute psychiatry [78,79], which should be further explored. This includes a close examination of the question of whether PAEHRs can contribute to suicide attempts or other violent behaviors. In this context, the subgroup of individuals that HCPs are most concerned about should be examined; that is SUs with whom it has been very difficult to develop trusting relationships, or are delusional or paranoid, or who are prone to violence. Few studies have focused on the impact of PAEHRs on psychotherapy. In addition, little is known about the SUs’ perceptions of what clinicians have written. In this context it has to be considered more closely which type of language is either helpful or hindering and which information should necessarily be included in the notes [31].

Fifth, further research should explore how PAEHRs affect documentation [80]. For example, it is not known whether access changes the quality of mental notes. For example, computer software programs that use validated metrics such as the Flesch-Kincaid reading scale could be employed to explore whether the length of notes changes after implementation or whether access changes readability.

Finally, as many of the included studies have mentioned concerns regarding privacy and data security [16,18,19,21,46,51], we think this theme has been covered rather superficially and the findings are sometimes contradictory. On the one hand, it was reported, for example, that SUs did not hold significant privacy concerns and worried more about data security [19], whereas in contrast privacy concerns were reported by both SUs and HCPs, which were partially reinforced after the implementation of a PAEHR [16]. Therefore, we believe that further, more rigorous, research on privacy and security is needed in the field of MHC.

### Strengths and Limitations of the Scoping Review

This is the first systematic scoping review collating existing evidence about sharing EHRs or clinical notes with people being treated for mental health conditions. Several limitations should be considered when interpreting the findings. On the one hand, our search may have missed some relevant studies owing to the variety of terms used for EHR (eg, clinical note, electronic medical record, and patient portal), and the restriction on English-language and peer-reviewed publications. However, by applying a rigorous search strategy and continuously expanding the search term in the process, 1032 potential records could be identified and screened, which, considering the relative novelty of the research subject, represents a comprehensive result. In addition, the review benefited from a diversity of authors, located in 4 countries (Germany, Norway, Sweden, and the United States), who bring a variety of academic and health care backgrounds to this exploration (psychiatric practice, implementation science, philosophy of medicine, and health care ethics). Although this work was not coproduced by researchers with and without lived experience of mental distress, it was critically reviewed and commented on by 2 user researchers (SM and LC) to ensure adequate engagement of the authors (JS, AB, CB, MH, and SH) with PAEHRs from an SU perspective.

### Conclusions

The corpus of evidence on sharing EHRs or clinical notes with people affected by mental health conditions is limited. Further research is needed to examine the clinical effects, costs, and implementation of PAEHRs. The user perspective on OpenNotes should be examined more closely with participatory design methodologies and involving researchers, including SUs and caregivers, with lived experience of mental distress.

## Appendix

Multimedia Appendix 1 PRISMA (Preferred Reporting Items for Systematic Reviews and Meta-Analyses) extension for Scoping Reviews Checklist.

Multimedia Appendix 2 Summary of included studies.

Multimedia Appendix 3 Mixed Methods Appraisal Tool ratings for each study.

Multimedia Appendix 4 Service user commentary.

## Abbreviations

EHR: electronic health record
HCP: health care professional
MHC: mental health care
MMAT: Mixed Methods Appraisal Tool
PAEHR: patient-accessible electronic health record
PRISMA: Preferred Reporting Items for Systematic Reviews and Meta-Analyses
PTSD: posttraumatic stress disorder
SU: service user
VA: Veterans Affairs
