# Supplementary material for: Sharing Clinical Notes and Electronic Health Records With People Affected by Mental Health Conditions: Scoping Review
Source: JMIR Ment Health. 2021 Dec 14;8(12):e34170. doi: 10.2196/34170 (PMC8715358; doi:10.2196/34170)
Supplement: Multimedia Appendix 2 [file mental_v8i12e34170_app2.docx]

**Multimedia Appendix 2.** Summary of included studies

| **Authors** | **Year** | **Ref.-ID** | **Country** | **Design** | **n** | **Participants** | **Setting** | **Purpose** | **Results** | **Quality** |
| --- | --- | --- | --- | --- | --- | --- | --- | --- | --- | --- |
| Adanijo et al. | 2021 | [[52]](https://www.zotero.org/google-docs/?uws8tp) | UK | qualitative | 22 | SUs | psychiatry | To investigate views of mental health SU on acceptable data sharing to provide clear recommendations for future data sharing systems. | Six key themes were identified, such as the purpose of data sharing - for public good, and continuity of care; discrimination through the misattribution of physical symptoms to mental health conditions, discrimination of individuals or groups within society; securing data by preserving anonymity; data accuracy and informed consent- increasing transparency about data use and choice; and incorporating SU involvement in system governance to provide insight and security. | ***** |
| Bärkås et al. | 2021 | [[51]](https://www.zotero.org/google-docs/?vgYGEM) | Sweden | qualitative | 24 | policy stakeholders | inpatient + outpatient psychiatry | To explore Swedish national and local policy regulations regarding PAEHRs and describe to what extent SU are offered access to notes. | The results show that 17 of 21 regions share psychiatric notes with SU, where forensic psychiatric care was the most excluded psychiatric care setting. All private care providers reported that, in the main, they follow the regions’ guidelines. The findings show that regional differences concerning sharing psychiatric notes persist, despite Swedish regulations and a national policy that stipulates equal care for everyone. | ***** |
| Blease et al. | 2021 | [[12]](https://www.zotero.org/google-docs/?ucD3gi) | USA | qualitative | 70 | policy stakeholders and experts | psychiatry | To solicit the views of experts on provider policies and SU and clinician training or guidance in relation to open notes in mental health care. | A total of 70 of 92 (76%) experts from 6 countries responded. Four major themes related to PAEHRs could be identified: I. the need for clarity about provider policies on exemptions, II. providing patients with basic information about PAEHRs, III. clinician training in writing mental health notes, IV. and managing patient-clinician disagreement about PAEHRs. | ***** |
| Blease et al. | 2021 | [[49]](https://www.zotero.org/google-docs/?79kbIK) | USA | quantitative | 19380 | SUs | outpatient setting across disciplines | To explore how SU with a mental illness diagnosis perceived how reading the note affected their adherence to prescribed medication. | Of the full sample, 7% had a diagnosis of SMI, and 9% had another mental illness. SU with SMI viewed their notes at a rate of 47% compared with SU without a mental health diagnosis, who viewed notes at a rate of. Among participants with an SMI diagnosis 20% reported that reading their notes made them more likely to take their medications, (67%) reported that reading notes helped them understand why medications were prescribed, (67%) felt more in control of their medications. | ***** |
| Chimowitz et al. | 2020 | [[18]](https://www.zotero.org/google-docs/?Tiiry1) | USA | qualitative | 9 | mental health social workers | outpatient psychotherapy | To explore attitudes and experiences of participating and nonparticipating therapists to PAEHRs including why they did or did not want to share therapy notes with SU. | Participating therapists stated PAEHRs could empower and inform SU and strengthen therapeutic relationships by (a) helping SU remember the session, (b) reassuring SU, (c) encouraging therapists and (d) improving patient-provider communication about concerns. Non-participating therapists raised concerns about threats to SU’ privacy, specifically about other providers seeing these notes. Most therapists would be more willing to share notes with patients if they could enable EHR protections. | ***** |
| Cromer et al. | 2017 | [[40]](https://www.zotero.org/google-docs/?Sf14hT) | USA | qualitative | 28 | veterans receiving mental health services | outpatient psychiatry (VA) | Investigation of the effects of PAEHRs on the patient-clinician relationships for veterans who receive mental health treatment. | SU felt the relationship with the clinician developed by establishing good rapport, talking openly and feeling comfortable with their clinicians. Trust in clinicians was strengthened on the basis of SUs’ evaluations of the levels of transparency and respect conveyed in notes. SU were more trusting when clinicians received diagnoses with them before documenting in their records. In summary, clinicians notes should be respectful and show the evidence of truly listening to their patients. | ***** |
| Denneson et al. | 2017 | [[15]](https://www.zotero.org/google-docs/?q8fwv3) | USA | qualitative | 28 | mental health clinicians, psychologists, social workers and nurses | outpatient psychiatry (VA) | Examination of mental health clinicians’ perspectives on and experiences with PAEHRs. | SUs’ access to their mental health information resulted in a shifting power distribution within the patient-clinician relationship. Progress notes could leave room for miscommunication and misinterpretation. In detail, PAEHRs are affecting how mental health clinicians build and maintain the therapeutic relationship and therapeutic process and mental health clinicians are adjusting their practices in the context of PAEHRs to protect SU and themselves from potential adverse consequences. | ***** |
| Denneson et al. | 2018 | [[41]](https://www.zotero.org/google-docs/?PzCWiq) | USA | quantitative | 178 | veterans receiving mental health care | outpatient psychiatry (VA) | This study investigated how reading PAEHRs changes mental health SU’ experiences and outcomes. | SU reported that PAEHRs helped them feel in control of their health care and have more trust in clinicians. Few SU experienced stress or worry after reading the notes, some stated that the notes make their problems seem smaller than they are. Education was associated with decreased patient-clinician alliance. PTSD was associated with increased patient-clinician alliance and positively associated with experiencing negative emotional responses to reading notes. | **** |
| Denneson et al. | 2019 | [[42]](https://www.zotero.org/google-docs/?ChHAIy) | USA | quantitative | 247 | veterans receiving mental health care | outpatient psychiatry (VA) | This study evaluates the outcomes of a web-based educational program on PAEHRs. | No significant change was observed in patient assessment of the therapeutic relationship. SU activation score and perceived efficacy in healthcare interactions increased significantly between the pre- and post- training. Older age and higher baseline eHealth literacy were significantly associated with a stronger therapeutic relationship. | ***** |
| Dobscha et al. | 2016 | [[13]](https://www.zotero.org/google-docs/?3h247Z) | USA | quantitative | 263 | mental health clinicians and nurses | outpatient psychiatry (VA) | To describe VA mental health clinician experiences with and attitudes toward OpenNotes use. | Half of the respondents agreed that PAEHRs were a good idea and will support SU to better remember their care plans. They also reported being less detailed and changing the tone of their notes. A minority reported using separate, i.e. hidden psychotherapy or process notes. Further, it was reported that they or SU had experienced specific negative consequences such as disengagement from care, disagreements about noted content, disruptions in the therapeutic relationships. | ***** |
| Dobscha et al. | 2018 | [[44]](https://www.zotero.org/google-docs/?UHcIeI) | USA | quantitative | 338 | veterans receiving mental health care | outpatient psychiatry (VA) | To Identify demographic, clinical, and knowledge-related predictors of viewing PAEHRs among SU who receive VA mental health care. | The majority of SU had been given a diagnosis of PTSD or depression (approx. 60%). Younger veterans were significantly more likely to have read their notes. There was a relationship between demographic variables and ever viewing notes: Highest education level and PTSD diagnosis were significantly associated with having viewed their notes. Moreover clinicians speaking with SU about being able to view their notes was significantly associated with SUs’ having read or downloaded notes. | ***** |
| Dobscha et al. | 2019 | [[43]](https://www.zotero.org/google-docs/?kDqRfG) | USA | quantitative | 251 | mental health clinicians | outpatient psychiatry (VA) | To report clinicians attitudes and patient-clinician communication behaviors related to use of PAEHRs after a web based course. | After attending the web based course, enrolled clinicians worried less about negative consequences of PAEHRs and their ability to communicate with and educate SU about PAEHRs increased. Clinicians advised SU more frequently to read their notes and asked them about questions or concerns they have with their notes after the course. A post hoc analysis of the data did not show a change in the overall results. | **** |
| Erlingsdóttir et al. | 2019 | [[16]](https://www.zotero.org/google-docs/?KrhliV) | Sweden | qualitative | 1554 | mental health care professionals | inpatient + outpatient psychiatry | To compare HCPs perceptions before and after implementation of a PAEHR to separate expectations from experiences. | HCPs reported being less candid and less effective when documenting in the PAEHR**.** They were concerned about SU discovering errors and SU interpretation of their notes and privacy of the notes. HCPs worried about losing their autonomy in how to write and control the notes. Many SU did not have the material resources to use PAEHRs. Implementation was not sufficiently prepared, and the enhanced transparency negatively affected the professionals’ work. | ***** |
| Etingen et al. | 2019 | [[53]](https://www.zotero.org/google-docs/?2EkH0v) | USA | quantitative | 159581 | veterans receiving mental health care | outpatient + inpatient psychiatry (VA) | To examine patient portal use among Veterans with mental health diagnoses. | SU who experienced military sexual trauma or had an anxiety disorder, post-traumatic stress disorder, or depression were associated with increased odds of portal use. SU with military sexual trauma reported having the highest use of all the portal features; medication refill, appointment view, secure messaging, and Blue Button. SU with bipolar disorder, substance use disorder, psychotic and adjustment disorders were associated with decreased odds of portal use. | ***** |
| Hilton et al. | 2012 | [[45]](https://www.zotero.org/google-docs/?1MuKFP) | USA | quantitative | 2871 | SUs with an HIV and mental health diagnosis | primary care setting | To determine if mental health condition is a barrier to engagement with web-based health information. | Among SU attending a safety-net clinic, 70% had a mental health condition or substance use disorder. The latter was not a barrier to engagement with web-based health information. Instead, the level of computer competency was useful for identifying individuals requiring substantial computer training in order to fully participate in the study. Intensive on-study training was insufficient to enable beginner computer users to complete study surveys. | *** |
| Jonnergård et al. | 2021 | [[56]](https://www.zotero.org/google-docs/?5OgBW4) | Sweden | Mixed method | 853 | mental health care professionals | inpatient + outpatient psychiatry | To investigate if belonging to a profession influences the choice of communication media and the perception of information when Open Notes is implemented. | The communication strategies as most important before the implementation were reported as education, meetings, email, and intranet pages. However, 49% of the HCPs reported receiving and absorbing information about the implementation at a workplace meeting, and 25% from informal conversations with colleagues. All HCPs reported “patient safety”, “information to HCPs”, and “HCPs participation in the process “as important for the implementation. | *** |
| Kariotis et al. | 2019 | [[21]](https://www.zotero.org/google-docs/?Tt1Nim) | Australia | qualitative | 11 | general medical practitioners (GPs) and psychologists | primary care setting | To explore the potential role, benefits and barriers of a personally controlled EHR in mental health care. | GPs’ experiences with PAEHRs encompass mental health information issues regarding sensitive data that might be accessed through PAEHRs. They were also concerned about the completeness of the PAEHR, as SU can choose what to share with specific clinicians and leave out certain important documents. Participants also reported barriers to PAEHRs use, including time burdens - due to additional documentation efforts -, and privacy and confidentiality concerns. | ***** |
| Kipping et al. | 2016 | [[57]](https://www.zotero.org/google-docs/?LbLiT7) | Canada | quantitative | 3590 | SUs with serious or persistent mental illness | inpatient + outpatient psychiatry | To conduct a benefits evaluation of a patient portal for SUs undergoing treatment for serious or persistent mental illness. | Over the year-long follow-up period after the portal implementation, the portal was used 4761 times by the SUs, with most logins for e-views. The implementation resulted in activation of SUs and improved recovery scores according to Mental Health Recovery Measure. The odds of SUs attending a scheduled appointment were 67% greater than that of nonusers. The administrative burden decreased as there was an 86% decrease in the number of requests for information among SUs. | ***** |
| Klein et al. | 2018 | [[50]](https://www.zotero.org/google-docs/?8xK0kn) | USA | quantitative | 2534 | SUs | primary care setting | To explore the potential impact of PAEHRs from the perspective of SU with or without a mental health diagnosis. | 400 out of 2534 participants had a mental health diagnosis. Most SU had positive perceptions about PAEHRs. SU with  mental health diagnoses were significantly more likely  to report worry compared with other patients, however, after adjustment for variables independently associated with mental health diagnoses (sex, race, education, and employment) there were no differences in perceptions  about access to online notes between patients with or without mental health diagnoses. | **** |
| Kristiansen et al. | 2019 | [[5]](https://www.zotero.org/google-docs/?nq3gWt) | Norway | quantitative | 6105 | (mental) health care professionals | all settings and medical disciplines | To investigate HCPs’ experience with PAEHRs some years after implementation. | A quarter of the HCPs noticed that patients were better engaged about their own health after online access to their EHR. Under 15% of the HCP used more time on explaining and calming SU after the implementation. However, one third of the HCPs spent more time on explaining and calming SU, and reported changing the way they wrote clinical notes. The results revealed that there were significant differences between the professions, regions and somatic and psychiatric healthcare. | ***** |
| Leung et al. | 2019 | [[46]](https://www.zotero.org/google-docs/?R27im1) | Canada | Mixed method | 110 | SUs and family members | inpatient + outpatient psychiatry | To identify SU’ and family readiness, needs and perceptions of a mental health portal. | SU were interested in accessing the portal for reasons related to convenience, availability and to check the accuracy of information in their record. They rated portal functions generally positively. Family members were interested in the portal because it would be helpful and convenient to facilitate their support of a family member with mental conditions. Less than a half of the SU were interested in providing portal access for family members, preferring to keep access private. | ** |
| Matthews, E. | 2020 | [[54]](https://www.zotero.org/google-docs/?VT1tQP) | USA | quantitative | 106 | mental health behavioral clinicians and SUs | outpatient psychotherapy | To evaluate the perceived impact of in-session computing on communication in mental health treatment from the SU and HCPs perspective. | 28% of the SU met the diagnosis criteria for serious mental illness (bipolar disorder, schizophrenia, or any type of psychotic disorder), with bipolar disorder as the most common (12.22%). SU (n=53) reported that computing frequency during visits did not impact the communication and increased the collaboration during planning visits. HCPs (n=53) reported perceived in-session computing as more harmful to communication and computer use as more disruptive than SU. | ***** |
| Matthews, E. | 2020 | [[55]](https://www.zotero.org/google-docs/?WZJszM) | USA | quantitative | 71 | mental health behavioral clinicians and SUs | outpatient psychotherapy | To explore collaborative documentation (CD) in psychotherapy, it’s implementation and effects on the therapeutic alliance. | 28.3% of the SU met the criteria for serious mental illness (any psychotic disorder and bipolar disorder). SU (n=58) reported that collaborative documentation (CD) endorsed a strong therapeutic relationship. HCPs (n=13) were highly likely to confirm the accuracy and acceptability of documentation with the SU. | ***** |
| O'Neill et al. | 2019 | [[47]](https://www.zotero.org/google-docs/?mwX9t7) | USA | Mixed method | 85 | SUs | outpatient psychotherapy | To understand SU attitudes about PAEHRs in psychotherapy and to explore if SU find PAEHRs beneficial to their mental health. | SU agreed that sharing therapy notes is a good idea, found notes easy to understand and made their decision for or against a therapist depending on the possibility to read EHRs. One-third read the notes to make sure that they were understood, some discussed the notes with their therapist. PAEHRs could increase trust in and openness with the therapist. Privacy concerns and feelings of being judged were raised. Some SU discovered incongruence between the notes and what occurred in the session. | ***** |
| Peck et al. | 2017 | [[19]](https://www.zotero.org/google-docs/?UGLio1) | USA | quantitative | 52 | SUs and clinicians | outpatient psychiatry | To understand the impact of PAEHRs on SU and clinicians in mental health care. | Through PAEHRs SU could make sure that they are on the same page as and were understood by the clinicians. SU reported that it was helpful to understand the visit from the providers’ point of view. SU reported concerns in cyber security and privacy. Clinicians agreed that note writing changed because of SU access, especially for SU with specific issues. Most clinicians felt strongly that SU be selected on a case by case basis. Clinicians perceived benefits in their communication with patients. | *** |
| Petersson et al. | 2018 | [[17]](https://www.zotero.org/google-docs/?O9mApg) | Sweden | quantitative | 871 | mental health care professionals | inpatient + outpatient psychiatry | To explore how different HCPs in adult psychiatric care expect PAEHRs to impact SU and their own practice. | More than a half of the HPCs believe that SU would worry more after reading their notes and that they would disagree with the content in their notes. About 30 % believed that PAEHRs will increase SU trust for them as professionals. In the HCPs’ view SU will not take better care of themselves, if they use PAEHRs. They also believed SU would find significant errors in the notes. | ***** |
| Petersson et al. | 2018 | [[14]](https://www.zotero.org/google-docs/?J5hB3V) | Sweden | quantitative | 691 | mental health care professionals | inpatient + outpatient psychiatry | To explore how mental HCPs expect PAEHRs to impact SU and their own practice and to compare results with a previously published baseline survey study. | HCPs generally thought that the expected benefits of PAEHRs for SU were somewhat absent so there was a little impact on practise. HCPs reported that they were less candid and spent more time editing notes. Few HCPs agreed that PAEHRs raised risks for threats and violence as well as it led to safer care. There were different opinions about PAEHRs between the professions. Less than a half of the nurses agreed that PAEHRs is a good idea. | ***** |
| Pisciotta et al. | 2019 | [[22]](https://www.zotero.org/google-docs/?TIhTPr) | USA | qualitative | 28 | mental health clinicians (VA) | outpatient psychiatry (VA) | To develop recommendations for mental health clinicians to help them effectively practice and write notes in a setting that provides PAEHRs. | HCPs stated notes should be professional and respectful, not contain spelling errors, inaccuracies nor should notes reuse information from the past notes. Open communication was felt to be especially important when documenting sensitive or potentially surprising information such as diagnoses. PAEHRs, it was recommended, present a unique opportunity for enhancing care by increasing opportunities for communication and collaboration. | ***** |
| Robotham et al. | 2015 | [[48]](https://www.zotero.org/google-docs/?QU5PdK) | UK | Mixed method | 58 | SUs | outpatient (+ inpatient) psychiatry | To explore how PAEHRs were used in practice, and how stakeholders thought they should be improved. | Less than a half of SU used the PAEHR. Usage did not differ by gender, by diagnosis or length of time using services. Most participants who used the site used it primarily or exclusively at the facilitated drop-ins. Most users said that they found the site useful and would continue to use it in the future. | *** |
| Strudwick et al. | 2018 | [[20]](https://www.zotero.org/google-docs/?Ngj774) | Canada | mixed methods | 250 | health care professionals (HCP) | inpatient + outpatient psychiatry | To identify predictors of mental HCPs’ perceptions of PAEHRs, change in documentation practice, and discomfort of patients accessing patient portals. | HCPs reported to have a more negative reception on patient portals than other HCP groups. Further, similar is true for HCPs working in acute care settings. The findings from the content analysis are in line with similar qualitative studies. Some HCPs are concerned about the readability of their notes for the patients and modifications introduced to their documentation when sharing notes with their patients. | ***** |
| van Rijt et al. | 2021 | [[23]](https://www.zotero.org/google-docs/?b1ijK0) | Netherlands | qualitative | 21 | mental health clinicians | inpatient + outpatient psychiatry across disciplines | To provide insights into the appraisal work of mental HCPs to assess and understand patient access to their EHRs through a patient portal. | HCPs reported being concerned that their treatment plans might no longer be effective due to patient access to their EHR. They also report real-time patient access, such as in situations when patients need acute care or are compelled to receive care, might lead to dangerous situations because patients act before mental HCPs can carry out their treatment plan. | ***** |

Abbreviations: EHR: Electronic Health Record; GP: General Practitioner; HCP: Health Care Professional; PAEHR: Patient-accessible electronic Health Record; SMI: Severe Mental Illness; SU: Service User; VA: Veterans Affairs.
