# Supplementary material for: Sharing Clinical Notes and Electronic Health Records With People Affected by Mental Health Conditions: Scoping Review
Source: JMIR Ment Health. 2021 Dec 14;8(12):e34170. doi: 10.2196/34170 (PMC8715358; doi:10.2196/34170)
Supplement: Multimedia Appendix 3 [file mental_v8i12e34170_app3.docx]

**Multimedia Appendix 3.** MMAT ratings for each study. (1…yes; 0…no; ?...can’t tell)

| **Studies** | | **Criteria from the Mixed Methods Appraisal Tool** | | | | | | | | | | | | | | | | | | | | | | | | |
| --- | --- | --- | --- | --- | --- | --- | --- | --- | --- | --- | --- | --- | --- | --- | --- | --- | --- | --- | --- | --- | --- | --- | --- | --- | --- | --- |
| **Authors** | **Ref.-ID** | 1.1 | 1.2 | 1.3 | 1.4 | 1.5 | 2.1 | 2.2 | 2.3 | 2.4 | 2.5 | 3.1 | 3.2 | 3.3 | 3.4 | 3.5 | 4.1 | 4.2 | 4.3 | 4.4 | 4.5 | 5.1 | 5.2 | 5.3 | 5.4 | 5.5 |
| Adanijo et al., 2021 | [[52]](https://www.zotero.org/google-docs/?f2N0BH) | 1 | 1 | 1 | 1 | 1 |  |  |  |  |  |  |  |  |  |  |  |  |  |  |  |  |  |  |  |  |
| Bärkås et al., 2021 | [[51]](https://www.zotero.org/google-docs/?Dzuyvi) | 1 | 1 | 1 | 1 | 1 |  |  |  |  |  |  |  |  |  |  |  |  |  |  |  |  |  |  |  |  |
| Blease et al., 2021a | [[12]](https://www.zotero.org/google-docs/?5UDbcN) | 1 | 1 | 1 | 1 | 1 |  |  |  |  |  |  |  |  |  |  |  |  |  |  |  |  |  |  |  |  |
| Blease al., 2021b | [[49]](https://www.zotero.org/google-docs/?cM1w1S) |  |  |  |  |  |  |  |  |  |  |  |  |  |  |  | 1 | 1 | 1 | 1 | 1 |  |  |  |  |  |
| Chimowitz et al., 2020 | [[18]](https://www.zotero.org/google-docs/?VQYXHF) | 1 | 1 | 1 | 1 | 1 |  |  |  |  |  |  |  |  |  |  |  |  |  |  |  |  |  |  |  |  |
| Cromer et al., 2017 | [[40]](https://www.zotero.org/google-docs/?W2A7WI) | 1 | 1 | 1 | 1 | 1 |  |  |  |  |  |  |  |  |  |  |  |  |  |  |  |  |  |  |  |  |
| Denneson et al., 2017 | [[15]](https://www.zotero.org/google-docs/?8sPRti) | 1 | 1 | 1 | 1 | 1 |  |  |  |  |  |  |  |  |  |  |  |  |  |  |  |  |  |  |  |  |
| Denneson et al., 2018 | [[41]](https://www.zotero.org/google-docs/?jfa2Qs) |  |  |  |  |  |  |  |  |  |  |  |  |  |  |  | 1 | 1 | 1 | ? | 1 |  |  |  |  |  |
| Denneson et al., 2019 | [[42]](https://www.zotero.org/google-docs/?xF00w9) |  |  |  |  |  |  |  |  |  |  | 1 | 1 | 1 | 1 | 1 |  |  |  |  |  |  |  |  |  |  |
| Dobscha et al., 2016 | [[13]](https://www.zotero.org/google-docs/?NY6BHR) |  |  |  |  |  |  |  |  |  |  |  |  |  |  |  | 1 | 1 | 1 | 1 | 1 |  |  |  |  |  |
| Dobscha et al., 2018 | [[44]](https://www.zotero.org/google-docs/?oqP3UR) |  |  |  |  |  |  |  |  |  |  |  |  |  |  |  | 1 | 1 | 1 | 1 | 1 |  |  |  |  |  |
| Dobscha et al., 2019 | [[43]](https://www.zotero.org/google-docs/?uGucpd) |  |  |  |  |  |  |  |  |  |  | 1 | 1 | 1 | ? | 1 |  |  |  |  |  |  |  |  |  |  |
| Erlingsdóttir et al., 2019 | [[16]](https://www.zotero.org/google-docs/?nIFd9D) | 1 | 1 | 1 | 1 | 1 |  |  |  |  |  |  |  |  |  |  |  |  |  |  |  |  |  |  |  |  |
| Etingen et al., 2019 | [[53]](https://www.zotero.org/google-docs/?cI49bI) |  |  |  |  |  |  |  |  |  |  | 1 | 1 | 1 | 1 | 1 |  |  |  |  |  |  |  |  |  |  |
| Hilton et al., 2012 | [[45]](https://www.zotero.org/google-docs/?NDk02f) |  |  |  |  |  | 1 | 0 | 1 | ? | 1 |  |  |  |  |  |  |  |  |  |  |  |  |  |  |  |
| Jonnergård et al. 2021 | [[56]](https://www.zotero.org/google-docs/?cATZL2) | 1 | 1 | ? | 1 | ? |  |  |  |  |  |  |  |  |  |  | 1 | 1 | 1 | 1 | 1 | 1 | 1 | 1 | 1 | ? |
| Kariotis et al., 2019 | [[21]](https://www.zotero.org/google-docs/?K7eRme) | 1 | 1 | 1 | 1 | 1 |  |  |  |  |  |  |  |  |  |  |  |  |  |  |  |  |  |  |  |  |
| Kipping et al., 2016 | [[57]](https://www.zotero.org/google-docs/?juC0pF) |  |  |  |  |  |  |  |  |  |  | 1 | 1 | 1 | 1 | 1 |  |  |  |  |  |  |  |  |  |  |
| Klein et al., 2018 | [[50]](https://www.zotero.org/google-docs/?LFUIQS) |  |  |  |  |  |  |  |  |  |  |  |  |  |  |  | ? | 1 | 1 | 1 | 1 |  |  |  |  |  |
| Kristiansen et al., 2019 | [[5]](https://www.zotero.org/google-docs/?SxXa9D) |  |  |  |  |  |  |  |  |  |  |  |  |  |  |  | 1 | 1 | 1 | 1 | 1 |  |  |  |  |  |
| Leung et al., 2019 | [[46]](https://www.zotero.org/google-docs/?4qpwOu) | 1 | 1 | 0 | 0 | 1 |  |  |  |  |  |  |  |  |  |  | 1 | 1 | 0 | 1 | 1 | 0 | 1 | 0 | 1 | 0 |
| Matthews et al., 2020a | [[54]](https://www.zotero.org/google-docs/?2IUoEw) |  |  |  |  |  |  |  |  |  |  |  |  |  |  |  | 1 | 1 | 1 | 1 | 1 |  |  |  |  |  |
| Matthews et al., 2020b | [[55]](https://www.zotero.org/google-docs/?T9VkDw) | 1 | 1 | 1 | 1 | 1 |  |  |  |  |  |  |  |  |  |  | 1 | 1 | 1 | 1 | 1 |  |  |  |  |  |
| O’Neill et al., 2019 | [[47]](https://www.zotero.org/google-docs/?jAtKQJ) | 1 | 1 | 1 | 1 | 1 |  |  |  |  |  |  |  |  |  |  | 1 | 1 | 1 | 1 | 1 | 1 | 1 | 1 | 1 | 1 |
| Peck et al., 2017 | [[19]](https://www.zotero.org/google-docs/?uI4Hd9) |  |  |  |  |  |  |  |  |  |  |  |  |  |  |  | 1 | 0 | 1 | ? | 1 |  |  |  |  |  |
| Petersson et al., 2018a | [[17]](https://www.zotero.org/google-docs/?yARF6S) |  |  |  |  |  |  |  |  |  |  |  |  |  |  |  | 1 | 1 | 1 | 1 | 1 |  |  |  |  |  |
| Petersson et al., 2018b | [[14]](https://www.zotero.org/google-docs/?ZKpgLY) |  |  |  |  |  |  |  |  |  |  |  |  |  |  |  | 1 | 1 | 1 | 1 | 1 |  |  |  |  |  |
| Pisciotta et al., 2018 | [[22]](https://www.zotero.org/google-docs/?PqLOLR) | 1 | 1 | 1 | 1 | 1 |  |  |  |  |  |  |  |  |  |  |  |  |  |  |  |  |  |  |  |  |
| Robotham et al., 2015 | [[48]](https://www.zotero.org/google-docs/?3YOoJh) | 0 | 0 | 1 | 1 | 1 |  |  |  |  |  |  |  |  |  |  | 1 | 1 | 1 | ? | 0 | 1 | 0 | 1 | 1 | 1 |
| Strudwick et al., 2018 | [[20]](https://www.zotero.org/google-docs/?zxjc1c) | 1 | 1 | 1 | 1 | 1 |  |  |  |  |  |  |  |  |  |  | 1 | 1 | 1 | 1 | 1 | 1 | 1 | 1 | 1 | 1 |
| van Rijt et al., 2021 | [[23]](https://www.zotero.org/google-docs/?PNQU7i) | 1 | 1 | 1 | 1 | 1 |  |  |  |  |  |  |  |  |  |  |  |  |  |  |  |  |  |  |  |  |
