# Supplementary material for: Sharing Clinical Notes and Electronic Health Records With People Affected by Mental Health Conditions: Scoping Review
Source: JMIR Ment Health. 2021 Dec 14;8(12):e34170. doi: 10.2196/34170 (PMC8715358; doi:10.2196/34170)
Supplement: Multimedia Appendix 4 [file mental_v8i12e34170_app4.docx]

#### Comment Prepared by *SM*

This paper focuses on the experiences, expectations, challenges, and research gaps associated with patient-accessible EHRs in MHC. Interestingly, a third of the research reviewed was conducted in Veterans Affairs Mental Health.

As a patient, it is good to learn that service users reported mainly positive experiences with PAEHRs, such as increased trust in their clinician, health literacy, and empowerment. I can also relate from my own experience to the negative experiences reported, which were related to inaccurate notes, disrespectful language used, or the uncovering of undiscussed diagnoses.

It is also interesting that for HCPs, concerns outweighed the benefits of sharing EHRs, including an increased clinical burden due to more documentation efforts and possible harm triggered by reading the notes. This also resonates with my experience of clinicians as being very averse to making an extra effort to support their patients and as being disproportionately risk averse.

Again, it was no surprise to me that relatives reported gaining a better understanding of their family members’ mental problems and were able to better support them when having access to their EHR. It is also positive from a pragmatic and ethical perspective that policy stakeholders recommend the development of guidelines and training for both clinicians and service users.

From a personal perspective, patients need to be able to access and contribute to their EHRs, to be able to access and share important information on their mental health, correct errors in the EHR, and empower themselves in the context of better managing their mental health.

As a mathematician, I also concur with the paper’s recommendation that these predominantly exploratory findings are quantitatively validated.

#### Comment Prepared by *LC*

This scoping review is an important and timely piece of work. In this comment, my perspective comes from being affected by mental health conditions that have accessed clinical notes. I assess how the review summarizes the effects of the patients’ opening health records.

I am particularly interested in reading this scoping review about sharing clinical notes with patients, because of my own long history of a complex set of mental health conditions. After a severe traumatic brain injury, I spent nearly 20 years of life as a revolving door patient, detained in various psychiatric institutions. I was given many different diagnoses, including anorexia nervosa, treatment-resistant schizophrenia, borderline personality disorder, and organic psychotic disorder. After a long, arduous journey, I found the help I needed and now I am fully recovered (although I still receive help from community mental health services).

At one point in my treatment, as an outpatient, I requested to see all my medical notes (from several different United Kingdom National Health Service Foundation Trusts). After some time, I received a large number of files, which filled a bookcase, as well as digital notes. I was overwhelmed by the quantity and substance of these records. Examining these notes felt like reading about someone else’s life (not mine). Crucial moments in my recovery were left out (or made unavailable). It seemed as though my history had been rewritten, not how I remembered it.

Reading my notes was shocking (did I really do that? Was I really like that?’ and disorientating (they missed a lot out; they did not understand; it does not make sense). However, this gave me a new perspective and proof. I experienced some very difficult moments in my treatment—some of these were left out of my notes, but the bookcase (and all the electronic records) added up. I could say: This is where I have been. I will never return there again.

In this way, reading my notes has been empowering and helpful—preventive. I can pigeon-hole these records and put them away (put my illness away).

As a result, it is interesting to read the results of this scoping review. The study covers a variety of attitudes toward accessing OpenNotes, although it does not include patients with eating disorders. It is a good idea to consider in more depth how OpenNotes can be made available to patients during inpatient treatment, and if people who are detained would find this helpful or detrimental. In my experience, if I had seen my notes while in a compulsory admission, it may have been overwhelming. I am not sure I was in a clear enough mental state to be able to understand what the doctors said about me. However, it could have offered me the opportunity to have my say. This could have prevented me from giving me the wrong care.

This review offers a summary of research on sharing health records with people affected by mental health conditions. The next step is to assess how seeing notes can improve patients’ recovery outcomes and revitalize care. Carefully presented coproduced care is the best way forward. This work should be conducted in direct collaboration with people with mental health conditions.
